# Fat, Muscle, and Bone Interactions in Obesity and the Metabolic Syndrome

**DOI:** 10.1155/2014/247076

**Published:** 2014-09-15

**Authors:** Reina Armamento-Villareal, Nicola Napoli, Debra Waters, Dennis Villareal

**Affiliations:** ^1^Baylor College of Medicine, Houston, TX 77030, USA; ^2^Michael E. DeBakey VA Medical Center, Houston, TX 77030, USA; ^3^Campus Biomedico, 00128 Rome, Italy; ^4^University of Otago, Dunedin 9054, New Zealand

The increasing numbers of individuals diagnosed with obesity have led to burgeoning health care cost directed not only at the accompanying metabolic abnormality but also on the care of the associated physical disability particularly in the elderly population [1, 2]. The latter has resulted in a growing interest in the interactions between fat, muscle, and bone in the context of obesity and modalities to address obesity-related limitations in physical function. Obesity and the metabolic syndrome are associated with fatty infiltration in the muscles which leads to poor muscle quality, poor strength, and poor physical function most especially in the elderly [3]. In addition, adipose tissue secretes inflammatory cytokines that may cause skeletal muscle inflammation and may contribute to poor muscle quality [4, 5]. Furthermore, for bone, the traditional concept that a high body weight is osteoprotective is now challenged by recent findings of an increased prevalence of fractures in obese patients [6–8]. It is possible that the increase in fractures in these patients could be related to frequent falls from physical frailty. However, recent scientific advances have shown that cell signals that promote mesenchymal stem cells to differentiate into the adipogenic pathway are associated with suppression of signaling in the myogenic and osteogenic pathways [9, 10], both detrimental to muscle and bone. Knowledge and understanding of these interactions have led to the development of animal models, identification of novel targets for possible new therapies, and development of modalities that may alleviate the negative consequences of obesity and the metabolic syndrome. Aside from improving the associated metabolic derangement, reversal of poor muscle function and prevention of fractures constitute the ultimate goal for care in these patients.

In this issue, the authors tackle this important relationship between muscle, fat, and bone in the context of obesity and the metabolic syndrome ranging from basic physiology to the more complex interrelationship of these organs in disorders related to excess body weight. In the MrOS study, although the incidence of fractures goes down as body mass index (BMI) goes up from normal to overweight and lower obese category, when adjusted for bone mineral density (BMD), an increase in the incidence of fractures has been reported as BMI increases further to the higher obese category [6]. In the animal study by H. Fehrendt et al., the authors showed that, compared to controls, animals fed a high fat diet have reduced cancellous bone mass, collagen expression, amount of osteoid, and cell-to-cell contacts, despite the absence of differences in BMD and the number of osteoblasts and osteoclasts. In addition, there was an increase in the number of apoptotic osteocytes in high fat diet mice compared to controls. Taken together, these findings provided some mechanistic insights into the increased risk of fractures associated with obesity even in the presence of adequate bone mass. The review paper by N. Napoli et al. also provided a pathophysiologic basis for diabetes mellitus-associated bone disease and the role of mesenchymal differentiation and the different circulating factors in the alteration in bone metabolism in these patients. Given the close association between obesity and type 2 diabetes, it would not be surprising if both shared the same bone findings.

The important relationship between vitamin D metabolism and obesity is addressed by the paper by C. Cipriani et al. In this article, the authors reviewed the different mechanisms for low circulating vitamin D levels in obese patients. Conversely, the article also presented data from reports raising the possibility that vitamin D could promote adiposity by enhancing adipogenesis in low vitamin D states but not in vitamin D replete states. However, on the topic of vitamin D, an original report from N. Napoli and colleagues illustrated the altered bone turnover in patients with low vitamin D. Since hypovitaminosis D is common in obese patients and may contribute to the increased fracture prevalence reported in these patients, their findings underscored the need for vitamin D supplementation to normalize circulating vitamin D.

Frailty, which is common in obese older adults, is multifactorial and due to a combination of increased circulating adipokines (some of them proinflammatory) and poor muscle quality and from extra weight to carry with day to day activities. In the article by L. E. Aguirre et al., the authors demonstrated the individual and combined influence of the different hormones and cytokines on physical function among obese older adults. Among the circulating factors studied, it appears that testosterone, leptin, and adiponectin are important predictors of strength and endurance; however, TNF-α appears to be the only predictor of physical function. Along this line of research, O. Addison and colleagues reviewed the implications of increased intramuscular adipose tissue in metabolic, muscle, and mobility function. Increase in fatty deposition in the muscles contributes to poor muscle quality both from replacing muscles with fat and also from increased production of inflammatory cytokines from adipose tissues within the muscles. A review on the potential therapeutic targets and the effect of caloric restriction and exercise to mitigate fatty deposition in the skeletal muscles are also discussed in this article. There is evidence that lifestyle intervention by weight loss and exercise improves frailty in obese older adults, and although weight loss alone results in significant muscle and bone loss, the addition of exercise attenuates both muscle and bone loss [11]. The study by the group of G. Colaianni suggested that the myokine irisin may mediate the cross talk between muscle and bone. In their study, conditioned medium containing irisin from skeletal muscles of exercising animals induced a higher degree of osteoblastic differentiation compared to that coming from control animals. Aside from reenforcing the benefits of exercise on bone, it also suggests that anabolic effect of exercise on bone is mediated by the myokine irisin.

The degree of visceral fat accumulation in an individual may be dependent on the number of mitochondrial copies as suggested by the results of the study by J.-Y. Lee et al. Higher mitochondrial copy is associated with lower BMI, lower waist circumference, and reduced visceral fat emphasizing the genetic component of obesity.

There is evidence of an association between increasing BMI and susceptibility to musculoskeletal diseases in addition to frailty. The association between tendinopathies and obesity/diabetes was reviewed in the meta-analysis by the group of F. Franceschi et al. These authors analyzed the data from 15 papers showing higher odds ratios among obese individuals to develop tendinopathies. Another original study by U. G. Longo et al. investigated the relationship between high fibrinogen levels and rotator cuff injury (both of which are common in patients with metabolic disorders) but found no association. Finally, a study by U. Tarantino et al. evaluated the clinical and histomorphometric features of 80 patients undergoing hip arthroplasty for severe osteoarthritis (a common problem in obese patients) or osteoporosis-related femoral neck fractures. They found that bone volume fraction was lower in subjects with femoral neck fractures than in subjects with osteoarthritis and normal or osteopenic BMD. However, bone volume fraction in patients with combined osteoarthritis and osteoporosis is similar to that of patients with femoral neck fractures. The authors suggested that the limited mobility from hip osteoarthritis (likely severe) in some patients could contribute to the risk for developing osteoporosis.

The manuscripts in this issue highlight the interrelationships between fat, muscle, and bone in obesity and the metabolic syndrome and the disorders associated with derangement in this interaction. In this context, frailty appears to be a major consequence most especially in the elderly leading to loss of independence and increased nursing home admissions. Although the attainment of ideal body weight from lifestyle measures is highly unlikely in obese subjects, our group was able to show that a 10% weight loss with the addition of exercise improved physical function and attenuated the weight loss-associated muscle and bone loss [11]. Since not everyone is a candidate for lifestyle intervention, further studies are needed to identify potential targets to reverse frailty in the growing population of obese older adults.

*Reina Armamento-Villareal* *Reina Armamento-Villareal* *Nicola Napoli* *Nicola Napoli* *Debra Waters* *Debra Waters* *Dennis Villareal* *Dennis Villareal*

## References

[1] Finkelstein EA, Khavjou OA, Thompson H (2012). Obesity and severe obesity forecasts through 2030. *The American Journal of Preventive Medicine*.

[2] Lapane KL, Resnik L (2005). Obesity in nursing homes: an escalating problem. *Journal of the American Geriatrics Society*.

[3] Villareal DT, Banks M, Siener C, Sinacore DR, Klein S (2004). Physical frailty and body composition in obese elderly men and women. *Obesity Research*.

[4] Saghizadeh M, Ong JM, Garvey WT, Henry RR, Kern PA (1996). The expression of TNFα by human muscle: relationship to insulin resistance. *The Journal of Clinical Investigation*.

[5] Cesari M, Kritchevsky SB, Baumgartner RN (2005). Sarcopenia, obesity, and inflammation—results from the trial of angiotensin converting enzyme inhibition and novel cardiovascular risk factors study. *American Journal of Clinical Nutrition*.

[6] Nielson CM, Marshall LM, Adams AL (2011). BMI and fracture risk in older men: the osteoporotic fractures in men study (MrOS). *Journal of Bone and Mineral Research*.

[7] Compston JE, Watts NB, Chapurlat R (2011). Obesity is not protective against fracture in postmenopausal women: glow. *The American Journal of Medicine*.

[8] Premaor MO, Pilbrow L, Tonkin C, Parker RA, Compston J (2010). Obesity and fractures in postmenopausal women. *Journal of Bone and Mineral Research*.

[9] Bennett CN, Longo KA, Wright WS (2005). Regulation of osteoblastogenesis and bone mass by Wnt10b. *Proceedings of the National Academy of Sciences of the United States of America*.

[10] Christodoulides C, Lagathu C, Sethi JK, Vidal-Puig A (2009). Adipogenesis and WNT signalling. *Trends in Endocrinology & Metabolism*.

[11] Villareal DT, Chode S, Parimi N (2011). Weight loss, exercise, or both and physical function in obese older adults. *The New England Journal of Medicine*.

